# Correspondence

**Published:** 1866

**Authors:** 


### Correspondence.

PARIS, February 3d, 1866.

To the Editor of the Buffalo Medical and Surgical Journal :

*My Dear Doctor:*

At your request I will endeavor to post you in relation to my whereabouts, and furnish you with some hints upon medical matters in passing. Leaving New York on the morning of the 5th of January, upon the French steamer Europa, for Brest, we passed out through the narrows and commenced our voyage with a bright sun shining over us, and with light and favorable winds. On the 8th it commenced blowing, and the wind increased on the 9th and 10th to a gale, which continued with great severity until the 14th, tossing us about most wildly, and making most of the passengers severely sick. Although greatly retarded in our progress, the ship rode out the storm triumphantly. The English steamers London and Herschill, which encountered the same terrific gale, went down with nearly all their passengers on board. The influence of this tempest extended to all the eastern coasts of Europe, and across the Atlantic to our own shores, causing much loss of life and shipping everywhere. On the 15th, when only about five hundred miles from Brest, and when after escaping the great perils to which we had just been exposed, all were exchanging congratulations upon the favorable weather and our near approach to our destined port, and without apparent cause, the main shaft upon the port side suddenly broke completely in two. Instantly the engine upon the starboard side was smashed generally, in consequence of the breaking of the shaft upon the other side. Fortunately, or Providentially I should say, it was just the time for change of watch with the engineers. The chief was standing at the moment in a position which enabled him to arrest the engine, which he did instantly. But for this, another revolution would most likely have thrust a broken bar of iron through the bottom of the boat. The sea being now calm we were able to remove the floats or buckets from the wheels, and secure them (the wheels) from rotation, which the waves would have occasioned, and which must have torn the now broken engine in pieces and greatly endangered the boat. The vessel carried but little sail, and during the next thirty-six hours following the accident, she made at most five miles and some of the time only one and a quarter per hour, the wind being directly aft. Whilst this condition was highly favorable for making the broken engine secure it demonstrated our helplessness in case of head winds. The wind, though light, continued favorable when we approached Brest on the evening of the 18th, but we were unable to carry sufficient sail to make the port against the current made by the tide, which was at the time running out; the vessel was therefore headed for the channel. On Friday evening we were able to make the harbor of Cherbourg, the wind all this time continuing light and highly favorable. Within an hour after the steamer weighed anchor, it commenced blowing, and during the night increased to a gale. Had we encountered this gale, or had the weather at any time been rough whilst in our crippled condition, a kind Providence only could have saved us from destruction. In the narrow channel and in a vessel which could not be managed by her sails alone, we must have been driven on to the coast. With grateful hearts we disembarked on the 20th, but too happy to escape the numerous perils which we had encountered from the long terrific storm, and the still greater dangers from the accident to our machinery. Had the winds been adverse at any time during this period of four or five days we must have been driven out to sea, drifting about at their caprice and mercy. Had the storm of Friday night commenced but one hour earlier and before we were securely moored in Cherbourg, we must have been driven upon the nearest rocks. You can therefore well imagine that our hearts were filled with gratitude to Almighty God for his merciful preservation when we, safe in harbor, heard the gale of this night.

By means of this accident and our consequent necessity for taking shelter in Cherbourg we were afforded an opportunity of seeing the great naval sea-port of France. It is situated upon the narrowest part of the channel, and has been constructed, doubtless, at enormous expense, with reference to its proximity to England. The harbor was very unsafe until a large wall was built upon the seaward side. This wall, or *digue*, as it is called, is 4,111 yards (more than 2 miles) long, 120 yards broad at its base, and 16 at its summit, and no less than 22 yards high, occupying the open mouth of the bay, and leaving only sufficient space at either end for the ingress and egress of vessels. The fortifications upon this wall and the neighboring heights are formidable, but I do not believe they could resist Farragut and his modern American fleet for one hour.

We visited the dock yards and fortifications and were much interested in examining the *Trireme*, the only one doubtless of modern times. This, as you are aware, was constructed by the present Emperor, after the most ancient models, before writing his life of Julius Cæsar. The Trireme has arrangements for three banks of oarsmen, upon each side, sitting one above the other, extending the entire length of the vessel. This curiously constructed craft required 130 men for its propulsion. It is highly ornamented, much narrower and somewhat larger than one of our largest canal boats.

Leaving Cherbourg on Saturday evening we passed Sunday at Old Caen, and arrived in Paris on Monday evening. It may interest those who may chance to be in Caen to know that in addition to the many objects of curiosity which invite his attention there is a Protestant church with regular weekly services.

Many changes have taken place in this beautiful city (Paris) since I was here fifteen years since. But the secular journals have made your readers familiar with the great improvements effected by Napoleon III in the Boulevards and Parks, and I pass to a subject which will interest them more—the hospitals and medical men. Here also great changes are to be noted. Roux, who perhaps had performed more capital operations than any surgeon then living, being then more than seventy years old and very active, died some years since. He was constantly at his post in the wards of Hotel Dieu at that time, and a great favorite with all the *eleves.* Jobert, the most brilliant surgeon of that day in Paris, a thoroughly progressive man, has been compelled to abandon his post in consequence of mental imbecility, supposed to proceed from softening of the brain. Honored by the profession, decorated by and made the surgeon-in-chief to the Emperor, he has fallen a victim to *over work*—the uninterrupted strain upon the nervous system attending the vigorous pursuit of our profession. Although by no means an old man, he is in an asylum, and there likely to close his brilliant and promising career at no distant date. Velpeau, who, since the death of our own lamented Mott, and whose large work on surgery was translated by Mott, is probably the oldest living surgeon, and still retains his vigor. He is by no means regular in his attendance at the hospital, though little of importance transpires without his being consulted. Paul Dubois, who has for a long time occupied the front rank in the department of obstetrics, no longer gives his clinics or attends regularly at maternitè. Having been made a Baron, and having made himself rich by his immense practice, and by the large fees of the Empress and nobility, he now lives in dignified retirement, receiving patients for consultation but one or two hours daily. I regret to say that he is probably going to pass away without completing his work, long since commenced, upon his favorite department. The constant pressure of professional business has prevented his putting into permanent form the results of his reflections and immense experience, until he is now too old to render it probable that it will ever be accomplished.

Cazeaux, who stood second only to Dubois as a practitioner and teacher is also dead. Indeed the department of obstetrics has sustained many losses within a few years, in Paris, and I am fully prepared to endorse the remark made by an eminent practitioner a day or two since, that "the science of obstetrics had been stationary in this city during the last ten years." In uterine pathology and uterine surgery they have made greater advancement, but I sincerely believe from the limited opportunity thus far afforded of judging, that quite as much progress has been made in America in the diagnosis and treatment of all the forms of uterine disease during the last few years as in France.

Appropos to this subject I may inform you that our own countryman, Sims, remains without a competitor in his speciality, both in Paris and London. A collection of his monographs with large additional notes upon uterine surgery and sterility has just been published in London, and will soon be issued in New York by the Woods. Through the generosity of the author I have received a copy of the work, which is neatly got up by Churchill, the great London medical publisher, making an octavo volume of about five hundred pages. I have to some extent looked over its contents, and find much to approve. The surgical operations of which Dr. Sims is the father, are fully described, with all the improvements which his great experience in their performance has enabled him to suggest. This work, whilst it is by no means confined to the subject of vesico-vaginal fistula, does not profess to be a *complete* treatise upon uterine disease. Sterility is freely discussed and its treatment indicated when it depends upon displacement, flexion, stricture of the canal of the neck, or other deformities, and the author's views of treatment given. There is a very common source of sterility which is scarcely alluded to in this volume, and which has received little notice from writers and practitioners, which, if time permitted, I would like to discuss more fully. I allude to the plugging up of the uterine outlet by a gelatinous secretion of the glands of the cavity of the neck. It often attends endomitritis, and we find the os so completely closed by this tenacious gelatiniform secretion that it is very difficult to remove it by wiping with cotton; it must completely *tampon* the canal of the neck. You will remember doubtless that I have more than once referred to this subject in the City Medical Association, and when time serves may inflict my views upon you more at length.

As I am off in the morning for Spain I must close this rambling communication, merely commending the work of Dr. Sims to your favorable notice, which in my opinion it certainly merits.

Never forgetful of home friends I remain

Truly yours,

James P. White.

---

Utica, February 19, 1866.

To the Editor of the Buffalo Medical and Surgical Journal:

Dear Doctor:—I send you the following case, which you will publish if you see fit:

On the 25th of January last I was called to see Mr. D. R., aged 77. I learned that on the 23d he had been exposed to severe cold, and on the evening of the 24th he had been seized with severe and repeated rigors. He called a physician who said he had colic. He had not urinated for twenty-four hours, and for three days he had passed but a small quantity of dark urine; he had no difficulty in micturition. He had pain in the renal region, but more severe in the umbilical, which was aggravated by any movement of the body. His pulse was 104; tongue coated with a yellowish fur; nausea; no appetite; skin dry; bowels constipated; he was dull, probably insensible. There was no urine in the bladder. Confident that the patient had acute suppression of urine, (instead of colic,) and unless speedily relieved would die of uræmic poisoning, immediately called Dr. Bagg in consultation, who agreed with my diagnosis. Ordered a large mustard poultice over the umbilical and lumbar regions; gave calomel gr. xv, and ordered acetate of potassa 20 gr. in solution every two hours. Four o'clock P. M. gave fl. ext. senna ℥ss; 9 o'clock P. M. ordered an enema. 26th, 8 A. M. had had three evacuations; had passed ℥iv of bloody urine; pulse 100; tongue more coated. Ordered to continue the potassa and use the mustard as before. Four o'clock P. M. Dr. Bagg in consultation; had passed since morning ℥iv of dark urine; had vomited; had had a chill. Ordered a powder of calomel and ipecac āā, gr. ss, to be alternated every four hours with the potassa. 27th, no change; continued the potassa. 28th, had passed Oij of clear urine; pulse 92; tongue heavily coated; gave calomel gr. v, and continued the potassa. 29th, same; treatment same. 30th, convalescing; bowels had acted; tongue cleaning; urine natural in quality and quantity. Ordered citrate of iron gr. v, in solution, to be alternated every six hours with grs. x of the potassa. He continued to convalesce so that he was about the house February 5th, and on the 10th walked out.

IRA D. HOPKINS, M. D.

---

DISEASED MEAT.—At the weekly meeting of the City Commissioners of Sewers, Dr. Letheby, the medical officer of health, reported that the sanitary officers had condemned 9090 lbs. of meat as unfit for human food; most of it was in a diseased condition, and 505 lbs. were from animals that had died from disease. He stated that last week there were 128 quarters of beef condemned in the city markets, and most of it was from animals affected with the cattle plague.—*London Lancet.*

